# Antioxidant Nanotherapies for the Treatment of Inflammatory Diseases

**DOI:** 10.3389/fbioe.2020.00200

**Published:** 2020-03-18

**Authors:** Chen-Wen Li, Lan-Lan Li, Sheng Chen, Jian-Xiang Zhang, Wan-Liang Lu

**Affiliations:** ^1^Department of Pharmaceutics, College of Pharmacy, Third Military Medical University, Chongqing, China; ^2^Department of Chemistry, College of Basic Medicine, Third Military Medical University, Chongqing, China; ^3^Department of Pediatrics, Southwest Hospital, Third Military Medical University, Chongqing, China; ^4^Department of Pharmaceutics, School of Pharmaceutical Sciences, Peking University, Beijing, China

**Keywords:** oxidative stress, nanoparticles, antioxidant nanotherapies, ROS, inflammatory diseases

## Abstract

Reactive oxygen species (ROS) are essential in regulating various physiological functions. However, overproduction of ROS is implicated in the pathogenesis of various inflammatory diseases. Antioxidant therapy has thus represented an effective strategy for the treatment of oxidative stress relevant inflammatory diseases. Conventional anti-oxidative agents showed limited *in vivo* effects owing to their non-specific distribution and low retention in disease sites. Over the past decades, significant achievements have been made in the development of antioxidant nanotherapies that exhibit multiple advantages such as excellent pharmacokinetics, stable anti-oxidative activity, and intrinsic ROS-scavenging properties. This review provides a comprehensive overview on recent advances in antioxidant nanotherapies, including ROS-scavenging inorganic nanoparticles, organic nanoparticles with intrinsic antioxidant activity, and drug-loaded anti-oxidant nanoparticles. We highlight the biomedical applications of antioxidant nanotherapies in the treatment of different inflammatory diseases, with an emphasis on inflammatory bowel disease, cardiovascular disease, and brain diseases. Current challenges and future perspectives to promote clinical translation of antioxidant nanotherapies are also briefly discussed.

## Introduction

Reactive oxygen species (ROS) are chemical species containing unpaired electrons of oxygen and oxidizing agents that are readily turned to free radicals, such as hydroxyl radical (•OH), superoxide anion radical (•${\text{O}}_{2}^{-}$), singlet oxygen (^1^O_2_), hydrogen peroxide (H_2_O_2_), and hypochlorous acid (HOCl) (Bayir, 2006; D'Autréaux and Toledano, 2007; Nosaka and Nosaka, 2017). Living organisms systematically keep a balance between ROS and antioxidant defenses including exogenous and endogenous antioxidants, such as vitamin C, vitamin E, glutathione, superoxide dismutase (SOD), catalase (CAT), and peroxiredoxins (PRXs). Basic levels of ROS play an essential role in the cellular signal transduction processes and maintain oxygen homeostasis in the physiological environment. However, excessive ROS can lead to oxidative stress, cause DNA fragmentation, protein oxidation, and lipid peroxidation, which are associated with various inflammatory diseases, such as cardiovascular diseases, inflammatory bowel disease (IBD), neurodegenerative diseases, asthma, diabetes, and arthritis (Andersen, 2004; Fraisl et al., 2009). Accordingly, antioxidant therapy has been recognized as a potentially plausible approach for treating ROS-related inflammatory diseases (Apostolova and Victor, 2015).

Over the past decades, a number of natural and synthetic anti-oxidative compounds have been reported. For example, Edaravone is the only clinically approved low molecular weight radical scavenger used for the treatment of cerebral ischemic stroke, which has been proved to exert its therapeutic effects through scavenging free radicals and upregulating endothelial NOS expression in the cerebral tissue, thus preventing lipid oxidation and reducing neuronal damage (Yamamoto et al., 1997; Edaravone Acute Infarction Study Group, 2003). Nevertheless, the clinical use of Edaravone has been limited due to its multiple adverse effects such as hepatic and renal toxicity (Watanabe et al., 2008). Various adverse effects of low molecular weight ROS scavengers are mainly due to their non-specific distribution, high renal clearance, and low delivery efficiency. The emergence of nanotechnology has considerably overcome these limitations and inspires the next wave of technological innovation in antioxidant therapy. Nanomedicines can remarkably improve the pharmacokinetics properties of antioxidative compounds and simultaneously decrease their side effects. More excitingly, some types of nanomaterials, such as inorganic nanoparticles, possess inherent antioxidant activities, by directly reacting with ROS and/or mimicking the natural antioxidant enzymes, thereby showing powerful ROS-scavenging capability. Various ROS-scavenging and/or ROS-responsive antioxidative nanotherapies derived from organic materials, or inorganic/organic hybrid materials have also attracted extensive attention recently (Lu et al., 2016; Saravanakumar et al., 2017), yielding promising results to decrease oxidative damages in various animal models of diverse diseases. In addition, gas-generating (e.g., hydrogen) nanomaterials showed beneficial therapeutic effects in inflammatory diseases, due to their anti-inflammatory and anti-oxidative properties (He et al., 2017; Zhang B. et al., 2019). In this context, different types of antioxidant nanotherapies have been constructed based on various materials, such as carbon, metal oxides, nanocrystals, lipids, and polymers, as well as a variety of newly developed materials (Petros and DeSimone, 2010).

Herein we aim to provide a comprehensive review on the recent progress in antioxidant nanoparticles for the treatment of inflammatory diseases. Different antioxidant nanoparticles based on inorganic and/or organic materials as well as drug-loaded nanoparticles are first introduced. Then we highlight biomedical applications of these nanoparticles in anti-inflammatory therapy, with emphasis on the treatment of IBD, cardiovascular disease, brain diseases, and other inflammatory diseases. Finally, major challenges regarding the clinical translation of these antioxidant nanotherapies are discussed.

## Different Antioxidant Nanoparticles

### ROS-Scavenging Inorganic Nanoparticles

Over the past decades, a variety of inorganic nanoparticles based on carbon, metal, and metal-organic frameworks (MOFs) with natural enzyme-like activities have been developed to decrease the damage induced by ROS in biomedical areas (Table 1). These enzyme-like antioxidative nanomaterials have been defined as nanozymes by Yan in 2007 (Gao et al., 2007). ROS-scavenging inorganic nanoparticles show outstanding biological stability, versatile functionality, and regulatory activity.

**Table 1:** Representative ROS-scavenging inorganic nanoparticles.

**Nanoparticles**	**Applications**	**Evaluation models**	**Administration routes**	**References**
Fullerenes	Cortical infarction	*In vivo* in rats	Intravenous or intracerebroventricular injection	Lin et al., 2002
	Amyotrophic lateral sclerosis	*In vivo* in mice	Loaded into mini-osmotic pumps and implanted into the peritoneum	Dugan et al., 1997
	Alzheimer's disease	*In vivo* in rats	Intrahippocampal injection	Gordon et al., 2017
	Asthma	*In vitro* in human lung mast cells; *in vivo* in mice	Intranasal administration	Norton et al., 2012
Ceria oxide nanoparticles	Liver fibrosis	*In vivo* in rats	Intravenous injection	Oró et al., 2016
	Photoreceptor degeneration	*In vivo* in mice/rats	Intracardial injection; Intravitreal injection	Kong et al., 2011; Cai et al., 2012; Wong et al., 2013, 2015
	Alzheimer's disease	*In vivo* in mice	Unilateral subicular injection	Kwon et al., 2016
	Multiple sclerosis	*In vivo* in mice	Intravenous injection	Heckman et al., 2013
Platinum nanoparticles	Cerebral cavernous malformation disease	*In vitro* in HeLa, MCF-7, and Caco-2 cells	N/A	Moglianetti et al., 2016
	Cerebral ischemia	*In vivo* in mice	Intravenous injection	Takamiya et al., 2012
Manganese-based nanoparticles	Parkinson's Disease	*In vitro* in SH-SY5Y cells	N/A	Singh et al., 2017
	Ear-inflammation	*In vitro* in HeLa cells; *in vivo* in mice	Subcutaneous injection	Yao et al., 2018
Prussian blue nanoparticles	Liver inflammation	*In vitro* in HUVECs, RAW264.7, HBZY-1, NIH-3T3, and HT cells; *in vivo* in mice	Intravenous injection	Zhang W. et al., 2016
Molybdenum nanoparticles	Acute kidney injury	*In vitro* in HEK293 cells; *in vivo* in mice	Intravenous injection	Ni et al., 2018
V_2_O_5_@pDA@ MnO_2_ nanoparticles	Ear inflammation	*In vitro* in HEK293T cells; *in vivo* in mice	Intravenous injection	Huang et al., 2016
Ceria-zirconia nanoparticles	Sepsis	*In vitro* in RAW264.7 cells; *in vivo* in mice/rats	Intravenous injection; intraperitoneal administration	Soh et al., 2017

### Fullerenes

Fullerenes are the first reported SOD-mimicking inorganic nanomaterials, representing the landmark in the development of ROS-scavenging inorganic nanoparticles. Fullerenes are three-dimensional carbon networks, and the most common form of fullerenes is buckminsterfullerene (C_60_) with 60 carbon atoms arranged in a sphere. C_60_ fullerene can behave as a free radical “sponge,” with each C_60_ molecule capable of quenching multiple radical species (Krusic et al., 1991; Markovic and Trajkovic, 2008). The molecular mechanism underlying antioxidant reactions of C_60_ compounds is that electron-deficient regions of C_60_ cooperates with the attached malonyl groups to electrostatically guide and stabilize superoxide, thereby promoting its decomposition (Markovic and Trajkovic, 2008). C_60_(C(COOH)_2_)_2_ nanoparticles can be selectively internalized by oxidation-damaged cerebral endothelial cells, and greatly inhibit their apoptosis induced by ROS, which is related to the modulation of the c-Jun NH2-terminal kinase (JNK) signaling pathway (Lao et al., 2009). Several studies demonstrated that fullerenes can significantly alleviate ROS-dependent neuronal injury induced by N-methyl-D-aspartate or K^+^ deficiency *in vitro* (Dugan et al., 1997; Ali et al., 2008). The neuroprotective effects of fullerenes were also validated in cortical infarction induced by ischemia-reperfusion (Lin et al., 2002), amyotrophic lateral sclerosis in SOD mutant mice (Dugan et al., 1997), and Alzheimer's disease in rats induced by amyloid-β_25−35_ peptide (Gordon et al., 2017). Treatment with fullerenes also inhibited mast cell function *in vitro*, showing potent therapeutic effects on allergic asthma (Norton et al., 2012) and CCl_4_-mediated acute liver toxicity (Gharbi et al., 2005).

### Ceria Oxide Nanoparticles

**Figure 1:**
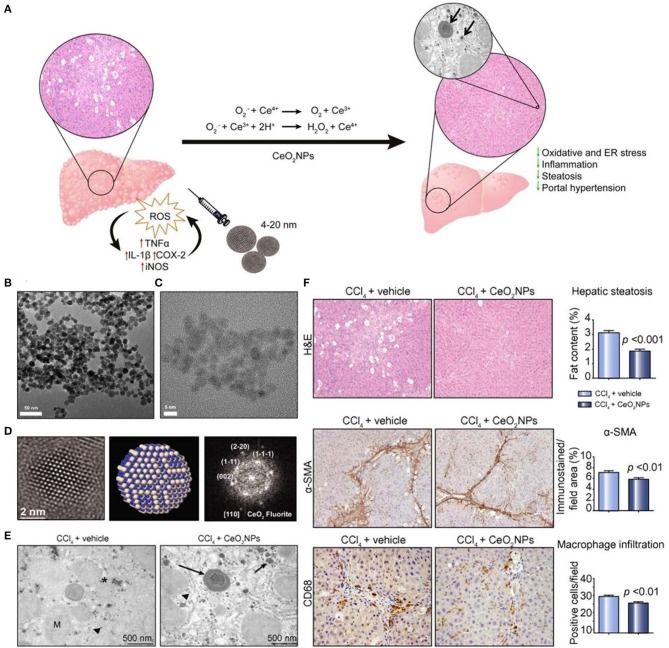
Schematic, characterization, and *in vivo* evaluation of CeO_2_ nanoparticles (NPs). **(A)** Schematic illustration of therapeutic mechanisms of CeO_2_NPs. **(B)** High-resolution transmission electron microscopy (HR-TEM) photomicrograph at low (43,000×) magnification revealing loose CeO_2_NPs agglomerates. **(C)** High magnification image (400,000×) of individual 4 nm CeO_2_NPs with spherical shape. **(D)** (Left) The HR-TEM image of single CeO_2_ NP showing the spherical morphology; (middle) atomistic simulation of CeO_2_NP with a characteristic fluorite-like electronic structure; (right) FFT of the selected nanoparticle with indicated atomic planes. **(E)** TEM image of liver tissue obtained from a CCl_4_-treated rat receiving vehicle and a CCl_4_-treated rat receiving CeO_2_NPs. Hepatocyte intracellular space containing mitochondria (M), organelles, fat droplets (asterisk), glycogen inclusions (arrow head), and aggregates of CeO_2_NPs (arrows). **(F)** Effect of CeO_2_NPs on hepatic steatosis, fibrogenic and infiltrating cells. Reproduced with permission from Oró et al. (2016).

Ceria oxide nanoparticles have been demonstrated to possess SOD-mimetic activity, resulting from the mixed valence states of Ce^3+^ and Ce^4+^ (Celardo et al., 2011; Bryant et al., 2016). It has become the most widely used inorganic nanomedicine for antioxidant therapy. At the nanoscale, Ce^3+^ and Ce^4+^ coexist on the surface of the Ce oxide lattice, and Ce^4+^ can be reduced to Ce^3+^ due to the presence of surface oxygen vacancies, therefore effectively decreasing ROS levels. Previous studies suggested that the Ce^3+^/Ce^4+^ ratio has an important effect on the antioxidant activity of ceria oxide nanoparticles, which is dominated by the microenvironment (Das et al., 2013). Li et al. reported a novel strategy to significantly improve the superoxide-scavenging activity of ceria oxide nanoparticles (> 5 nm) with different shapes and a negligible Ce^3+^/Ce^4+^ ratio through electron transfer (Li et al., 2015). Cerium oxide nanoparticles exhibited tissue protective actions in inflammatory diseases (Hirst et al., 2009), wound healing (Davan et al., 2012), photoreceptor degeneration (Cai et al., 2012), and neurodegenerative diseases (Kwon et al., 2016). Also, cerium oxide nanoparticles can accumulate in the liver and spleen following intravenous (i.v.) injection, showing effectiveness in therapy of liver fibrosis in rats (Oró et al., 2016). Mechanistically, cerium oxide nanoparticles are able to reduce ROS levels in HepG2 cells and protect CCl_4_-treated rats against chronic liver injury by inhibiting liver steatosis, reducing portal hypertension, and decreasing overexpression of inflammatory genes (Figure 1).

### Platinum Nanoparticles

Platinum is mainly used as a chemotherapeutic agent in clinical practice. Platinum nanoparticles were proven to have multiple activities mimicking SOD, catalase (CAT), and NADPH oxidoreductase (Hikosaka et al., 2008; Zhang et al., 2010; Oró et al., 2016; Pedone et al., 2017; Francesca et al., 2018), and they can catalyze the reduction of H_2_O_2_ to water and molecular oxygen, suggesting that platinum nanoparticles are potential medicinal candidates for oxidative stress diseases. Platinum nanoparticles possess strong and broad antioxidant properties, and they could significantly decrease the lipopolysaccharide (LPS)-induced production of intracellular ROS and inflammatory cytokines *in vitro* (Rehman et al., 2012). As radical scavenging materials, platinum nanoparticles showed potent activity in a cellular model of human cerebral cavernous malformation disease (Moglianetti et al., 2016), exhibited significant neuroprotective effects on ischemic mouse brains (Takamiya et al., 2012), effectively protected keratinocytes against UV-induced inflammation (Yoshihisa et al., 2010), and inhibited pulmonary inflammation induced by acute cigarette smoking (Onizawa et al., 2009). Kim et al. conjugated platinum nanoparticles with a fusion protein based on a platinum binding peptide and a HIV-1 TAT-derived peptide. Of note, the TAT peptide is a cell-penetrating peptide capable of enhancing translocation and internalization of platinum nanoparticles into the cytoplasm. Compared with unconjugated platinum nanoparticles, the fusion protein-conjugated platinum nanoparticles drastically improved the bioavailability of platinum nanoparticles in *Caenorhabditis elegans*, leading to similar antioxidant effects at only one hundredth dose (Kim et al., 2010).

### Manganese-Based Nanoparticles

**Figure 2:**
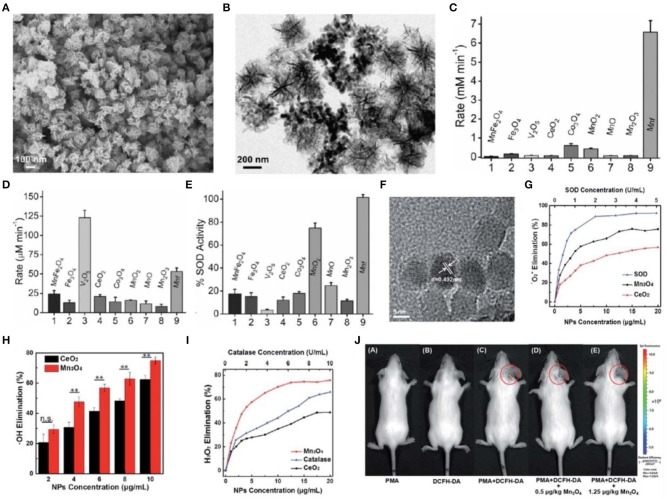
Characterization and antioxidative activity of manganese-based nanoparticles. **(A,B)** Scanning electron microscopy (SEM) **(A)** and TEM **(B)** images of Mn_3_O_4_ nanoparticles with flower-like morphology (Mnf). **(C–E)** A comparison of CAT **(C)**, glutathione peroxidase **(D)**, and SOD **(E)**-like activity of Mnf with other metal oxide nanoparticles. **(F)** TEM image of Mn_3_O_4_ nanoparticles synthesized via a hydrothermal method. **(G–I)** Dependence between the elimination of •O^2−^ **(G)**, •OH **(H)**, H_2_O_2_ **(I)** and concentrations of Mn_3_O_4_ and CeO_2_ nanoparticles. **(J)** *In vivo* fluorescence imaging of mice with PMA-induced ear inflammation after treatment with Mn_3_O_4_ nanoparticles. Images in **(A–E)** are reproduced with permission from Singh et al. (2017). Images in **(F–J)** are reproduced with permission from Yao et al. (2018).

A previous study demonstrated that natural MnSOD is superior to FeSOD and Cu/Zn SOD for the treatment of oxidative stress-related chronic diseases (Miriyala et al., 2012). This implicated that manganese-based nanoparticles may possess excellent ROS-scavenging activities (Singh et al., 2018; Singh N. et al., 2019). A recent study reported the flower-like Mn_3_O_4_ nanoparticles that functionally mimic three major antioxidant enzymes including SOD, CAT, and glutathione peroxidase (GPx). Mn_3_O_4_ nanoparticles exhibited excellent antioxidant activities and played a crucial role in protecting Parkinson disease-like cells from cytotoxicity induced by a neurotoxin 1-methyl-4-phenylpyridinium (MPP+) *in vitro* (Singh et al., 2017) (Figures 2A–E). In another study, Mn_3_O_4_ nanoparticles were synthesized via a hydrothermal method (Sun et al., 2019). These nanoparticles scavenged nearly 75% •${\text{O}}_{2}^{-}$ due to the two oxidation states of Mn^2+^ and Mn^3+^, which is superior to CeO_2_ nanoparticles. In addition, Mn_3_O_4_ nanoparticles can eliminate H_2_O_2_ and •OH. They demonstrated intracellular ROS-scavenging activities in HeLa cells *in vitro* and effectively protected mice from ROS-induced ear-inflammation *in vivo* (Figures 2F–J).

### Other ROS-Scavenging Inorganic Nanoparticles

In addition to the above mentioned nanoparticles, some other inorganic nanoparticles have been examined for ROS-scavenging, such as nanoparticles derived from Prussian blue (Zhang W. et al., 2016; Chen et al., 2018), gold/platinum and diamond (Martín et al., 2010), ruthenium (Cao et al., 2017), molybdenum (Ni et al., 2018), and vanadium (Vernekar et al., 2014). In particular, Zhang et al. discovered that Prussian blue nanoparticles possess CAT- and SOD-like activities, thereby effectively scavenging ROS, protecting cells from oxidative stress induced by cisplatin, diallyl trisulfide, LPS, phorbol 12-myristate 13-acetate (PMA), UV irradiation, high-level glucose, oxidized low density lipoprotein (OxLDL), and hypoxia/hypoglycemia and reoxygenation *in vitro* (Zhang W. et al., 2016). Prussian blue nanoparticles also exerted desirable anti-inflammatory effects in LPS-induced liver inflammation in mice. The peroxidase-like properties of Prussian blue nanoparticles are mainly ascribed to the FeN_*x*_ units (Chen et al., 2018). Ruthenium nanoparticles could also decompose H_2_O_2_ and scavenge •OH, •${\text{O}}_{2}^{-}$, ^1^O_2_, 2,2′-azino-bis(3-ethylbenzothiazoline-6-sulfonic acid-derived free radicals (ABTS•^+^), and 1,1-diphenyl-2-picrylhydrazyl radical (•DPPH), and therefore exert cytoprotective effects against H_2_O_2_-induced oxidative stress *in vitro* (Cao et al., 2017). However, *in vivo* efficacies of ruthenium nanoparticles remain to be explored. In another study, Mugesh et al. found that V_2_O_5_ nanowires displayed notable GPx-like antioxidant activity to protect cells from oxidative damage (Vernekar et al., 2014). Ceria-zirconia composite nanozymes (Soh et al., 2017), Gd@C_82_-(ethylenediamine)_8_ nanoparticles (Li et al., 2016), and V_2_O_5_@pDA@MnO_2_ nanocomposites (Huang et al., 2016) with multiple antioxidant activities were also investigated.

### Organic Nanoparticles With Intrinsic Antioxidant Activities

Besides ROS-scavenging inorganic nanoparticles, organic nanoparticles with intrinsic antioxidant activities have been emerging as a potential platform for the treatment of numerous diseases associated with inflammatory disorders (Kim K. S. et al., 2015; Zhu and Su, 2017; Kwon et al., 2019). Several types of organic nanoparticles have shown higher antioxidative advantages over conventional antioxidants and radical scavengers, owing to their predominant properties in terms of pharmacokinetics, biodistribution, delivery efficiency, and robust scavenging capabilities of multiple radicals (Lu et al., 2016; Ferreira et al., 2018; Ye et al., 2019; Zafar et al., 2019). In this section, these organic nanoparticles will be briefly introduced (Table 2).

**Table 2:** Different organic nanoparticles with intrinsic antioxidant activities.

**Types of organic nanoparticles**		**Applications**	**Evaluation models**	**Administration routes**	**References**
Bilirubin-derived nanoparticles	BRNPs	Hepatic ischemia-reperfusion injury	*In vitro* in primary mouse hepatocytes; *in vivo* in mice	Intravenous injection	Kim J. Y. et al., 2017
		Asthma	*In vivo* in mice	Intravenous injection; intraperitoneal administration	Kim D. E. et al., 2017
		Inflammatory bowel disease	*In vivo* in mice	Intravenous injection	Lee et al., 2016a
		Pancreatic islet xenotransplantation	*In vivo* in mice	Intraperitoneal administration	Kim M. J. et al., 2017
	DOX@BRNPs Dox@bt-BRNPs cisPt@BRNPs	Tumor	*In vivo* in mice	Intravenous injection	Lee et al., 2016b, 2017, 2018
	HABN	Colitis	*In vivo* in mice	Oral administration	Lee et al., 2020
Melanin-like nanoparticles	MMPP	Acute kidney injury	*In vitro* in HEK293 cells; *in vivo* in mice	Intravenous injection	Sun et al., 2019
	PEG-MeNPs	Ischemic stroke	*In vitro* in Neuro 2A cells; *in vivo* in mice	Intravenous injection	Liu et al., 2017
	PDA-PEG/CP	Tumor	*In vitro* in HeLa cells	N/A	Zhu and Su, 2017
	Liposome-BSM	Tumor	*In vivo* in mice	Subcutaneous injection	Chu et al., 2016
	PDA NPs	Periodontal disease	*In vivo* in mice	Subgingival administration	Bao et al., 2018
	PDA	Acute peritonitis and acute lung injury	*In vivo* in mice	Intravenous injection; intraperitoneal administration	Zhao et al., 2018
Free radical-containing organic nanoparticles	RNP^N^ and RNP^O^	Breast cancer	*In vitro* in MDA-MB-231 cells; *in vivo* in mice	Intravenous injection	Shashni and Nagasaki, 2018
	RNP^O^	Inflammatory bowel disease	*In vivo* in mice	Oral administration	Vong et al., 2012, 2014, 2015
	RNP^N^ and RNP^O^	Colon cancer	*In vitro* in C-26 cells; *in vivo* in mice	Intravenous injection	Vong and Nagasaki, 2016
	RNP^N^ and RNP^O^	Epidermoid cancers	*In vitro* in KB-3-1 and KB/MRP cells	N/A	Shashni et al., 2017
	RNP^N^	Radiation-induced organ dysfunctions	*In vitro* in RAW264.7 cells; *in vivo* in mice	Subcutaneous injection	Feliciano et al., 2017
	RNP^N^	Ischemia reperfusion injury	*In vivo* in mice	Intravenous injection	Yoshitomi et al., 2011
	RNPs	Nonalcoholic steatohepatitis	*In vivo* in mice	Intravenous injection	Eguchi et al., 2015
	RNPs	Neurological deficits	*In vivo* in mice	Intravenous injection	Chonpathompikunlert et al., 2012
Phenolic nanoparticles	HPOX	Asthma	*In vitro* in RAW264.7 cells; *In vivo* in mice	Intratracheal administration	Yoo et al., 2013
		Hindlimb ischemia	*In vivo* in mice	Intramuscular injection	Cho et al., 2015
		H_2_O_2_-associated inflammatory diseases	*In vitro* in RAW264.7 cells	N/A	Cho et al., 2012
	PVAX	Acute liver failure	*In vitro* in RAW264.7 cells; *in vivo* in mice	Intravenous injection	Ko et al., 2014
	PVO	Hepatic ischemia-reperfusion injure	*In vivo* in mice	Intravenous injection	Kang et al., 2016
Nanoparticles derived from functional cyclodextrin materials	Nanoparticles based on PBAP-conjugated cyclodextrin	Tumor	*In vitro* in B16F10 and RAW264.7 cells; *in vivo* in mice	Intravenous injection	Zhang et al., 2015
		Arterial restenosis	*In vitro* in rat VSMCs; *in vivo* in rats	Intravenous injection	Feng et al., 2016
		Inflammatory bowel disease	*In vivo* in mice	Oral administration	Zhang Q. et al., 2016
		Atherosclerosis	*In vitro* in RAW264.7 cells; *in vivo* in mice	Intravenous injection	Dou et al., 2017
		Abdominal aortic aneurysm	*In vivo* in mice	Intravenous injection	Cheng et al., 2018
		Peritonitis	*In vitro* in RAW264.7 cells; *in vivo* in mice	Intraperitoneal administration	Zhang et al., 2017
		Colitis-associated colon cancer	*In vitro* in C26 murine colon carcinoma cells; *in vivo* in mice	Oral administration	Zhang Q. et al., 2019
	LCD nanoparticles	Peritonitis, acute lung injury, and atherosclerosis	*In vitro* in neutrophils and macrophages; *in vivo* in mice	Intraperitoneal administration; intravenous injection	Guo et al., 2019
	TPCD nanoparticles	Peritonitis, acute lung injury, drug-induced organ toxicity, and atherosclerosis	*In vivo* in mice	Intraperitoneal administration; intravenous injection	Li et al., 2018; Wang et al., 2018
Other organic nanoparticles	PVA-C	Air pouch model	*In vitro* in BMSCs; *in vivo* in rats	Subcutaneous injection	Wu et al., 2019

### Bilirubin-Derived Nanoparticles

Bilirubin, a natural metabolite of hemoglobin, has been suggested as a primary physiological antioxidant for scavenging various ROS and protecting cells/tissues from oxidative damage. However, clinical applications of bilirubin have been restricted due to its poor water-solubility and toxicity. To overcome the drawbacks of natural bilirubin, Jon and coworkers developed polyethylene glycol (PEG)-conjugated bilirubin nanoparticles through a combined covalent conjugation and self-assembly method, which were defined as BRNPs (Lee et al., 2016a; Kim D. E. et al., 2017; Kim J. Y. et al., 2017). Compared to pristine bilirubin, BRNPs have more excellent water dispersibility, better pharmacokinetic properties, and higher accumulation in oxidative stress-induced inflammatory lesions. More importantly, BRNPs inherited the intrinsic powerful antioxidant capability of bilirubin, as evidenced by their efficacies in the treatment of a variety of oxidative stress-associated diseases, such as hepatic ischemia-reperfusion injury (Kim J. Y. et al., 2017), asthma (Kim D. E. et al., 2017), IBD (Lee et al., 2016a), and pancreatic islet xenotransplantation (Kim M. J. et al., 2017). In addition, BRNPs protected different cells against cytotoxicity caused by H_2_O_2_ and decreased inflammatory responses resulting from activated macrophages. Moreover, other bilirubin-derived nanoparticles can be used as a companion medicine for effective treatment of cancers and colitis (Lee et al., 2017, 2018, 2020). As a typical paradigm, Lee et al. constructed a hyaluronic acid-bilirubin nanomedicine (HABN) for inhibition of acute colitis (Lee et al., 2020). *In vivo* experiments demonstrated that HABN could target the inflamed colonic epithelium, restore the dysregulated intestinal barriers, and simultaneously modulate the gut microbiota and immune responses in mice bearing colitis.

### Melanin-Like Nanoparticles

Melanins are well-known endogenous biopolymers that are widely distributed in most living organisms. They have attracted much attention because of their fascinating biological characteristics, such as free radical quenching, photoprotection, and photosensitization (Ju et al., 2011; Solano, 2017; Wang X. et al., 2019). However, melanins can only be dissolved in strongly alkaline aqueous solutions, which largely limits their biomedical applications (Wang X. et al., 2019). Bioinspired polymerization of dopamine can generate melanin-like nanoparticles with similar properties to natural melanins. These melanin-like nanoparticles, often known as polydopamine (PDA) nanoparticles, contain a population of antioxidant groups, thereby exhibiting strong free radical-scavenging ability. PDA nanoparticles have been widely studied in recent years (Chu et al., 2016; Liu et al., 2017; Zhu and Su, 2017; Bao et al., 2018; Zhao et al., 2018; Sun et al., 2019). Cai et al. designed a melanin-based antioxidant defense nanosystem for acute kidney injury (Sun et al., 2019). The ultrasmall Mn^2+^-chelated and PEG-decorated melanin (MMPP) nanoparticles exhibited good physiological stability and excellent anti-oxidative activities toward various toxic ROS. *In vivo* results indicated that MMPP nanoparticles can serve as a multifunctional nanotheranostic system for the treatment of acute kidney injury. Notably, Shi's proved that bioinspired melanin nanoparticles (MeNPs) have excellent scavenging activity for multiple reactive oxygen and nitrogen species (RONS), such as •${\text{O}}_{2}^{-}$, H_2_O_2_, •OH, •NO, and ONOO^−^ (Liu et al., 2017).

### Free Radical-Containing Organic Nanoparticles

Spurred by the development of enzymology, a series of ROS-scavenging organic nanoparticles with enzyme-mimicking functions have been engineered as new antioxidant candidates for the biomedical applications. As a representative example, antioxidative nanotherapies self-assembled by integrating a stable nitroxide radical-containing ROS trapper, 4-hydroxy-2,2,6,6-tetramethylpiperidin-1-oxyl (Tempol) into the side chain of amphiphilic copolymers have been intensively investigated by Nagasaki's group (Yoshitomi and Nagasaki, 2014; Ikeda and Nagasaki, 2018). Two types of redox nanoparticles (RNPs), pH-sensitive RNP^N^ and pH-insensitive RNP^O^ have been synthesized and demonstrated remarkable protective and therapeutic effects in diverse diseases, such as breast or colon cancers (Vong and Nagasaki, 2016; Shashni and Nagasaki, 2018), IBD (Vong et al., 2012, 2014, 2015), radiation-induced organ dysfunctions (Feliciano et al., 2017), ischemia reperfusion injury (Yoshitomi et al., 2011), non-alcoholic steatohepatitis (Eguchi et al., 2015), and neurological deficit (Chonpathompikunlert et al., 2012). These studies have unambiguously substantiated the antioxidative effects of RNP^N^ and RNP^O^ without discernible adverse side effects.

### Phenolic Nanoparticles

Phenolic compounds, such as hydroxybenzyl alcohol (HBA) and vanillyl alcohol have long been widely used for treating ischemic brain injury and coronary heart disease, because of their antioxidant, anti-inflammatory, and anti-nociceptive activities (Berwin Singh et al., 2018). Recently, HBA-containing copolyoxalate (HPOX) was designed and synthesized, which can be degraded completely to release therapeutic HBA and 1,4-cyclohexendimethanol in the presence of H_2_O_2_ (Yoo et al., 2013). The biocompatible HPOX nanoparticles exerted highly potent antioxidant and anti-inflammatory effects by reducing the generation of ROS and suppressing the expression of pro-inflammatory mediators in stimulated macrophages. Given their beneficial effects in a mouse model of asthma by suppressing the recruitment of eosinophils and neutrophils as well as the expression of iNOS, HPOX nanoparticles exhibited tremendous potential as an anti-asthmatic agent. Likewise, HPOX nanoparticles also significantly promoted angiogenesis and blood flow perfusion in a mouse mode of hindlimb ischemia (Cho et al., 2015). Through similar procedures, vanillyl alcohol-containing copolyoxalate (PVAX) and poly (vanillin oxalate) (PVO) were synthesized by a simple one-step polymerization, and both of them are able to scavenge H_2_O_2_ (Ko et al., 2014; Jeong et al., 2016; Jung et al., 2019). PVAX nanoparticles and manganese porphyrin showed synergistic antioxidant and anti-inflammatory activities in mice with acetaminophen-induced acute liver failure (Ko et al., 2014). Kang et al. developed H_2_O_2_-triggered bubble-generating antioxidant PVO polymeric nanomaterials that can significantly suppress liver damages due to ischemia/reperfusion injury by inhibiting inflammation and apoptosis (Kang et al., 2016). In view of the advantages of excellent biodegradability, antioxidant activity, anti-inflammatory property, and low cytotoxicity, these phenolic nanoparticles have great potential as therapeutics for inflammatory diseases.

### Nanoparticles Derived From Functional Cyclodextrin Materials

Most recently, our group has developed a series of ROS-responsive materials by conjugating phenylboronic acid pinacol ester (PBAP) onto β-cyclodextrin (β-CD), a cyclic oligosaccharide with excellent *in vivo* safety (Zhang and Ma, 2013). The obtained ROS-responsive materials can be used to construct nanoparticles for site-specific delivery and inflammation-responsive release of different therapeutics (Juni et al., 2013; Lamprecht, 2015; Zhang Q. et al., 2016; Alaarg et al., 2017; Kotla et al., 2019). Interestingly, nanoparticles based on PBAP-conjugated cyclodextrin materials can effectively eliminate H_2_O_2_, thereby inhibiting inflammatory responses and oxidative stress in stimulated macrophages (Zhang et al., 2017). These H_2_O_2_-eliminating cyclodextrin nanoparticles efficaciously alleviated the symptoms of peritonitis and colitis in mice, by reducing the counts of neutrophils and macrophages as well as inhibiting the secretion of pro-inflammatory cytokines, chemokines, and oxidative mediators (Zhang et al., 2017; Zhang Q. et al., 2019). Also, we found that nanoparticles prepared from a luminol-conjugated β-CD material (LCD) can inhibit inflammatory response, oxidative stress, and recruitment of neutrophils and macrophages (Guo et al., 2019). In addition, LCD nanoparticles effectively reduced the levels of tumor necrosis factor (TNF)-α, interleukin (IL)-1β, myeloperoxidase (MPO), and ROS in neutrophils stimulated with PMA.

**Figure 3:**
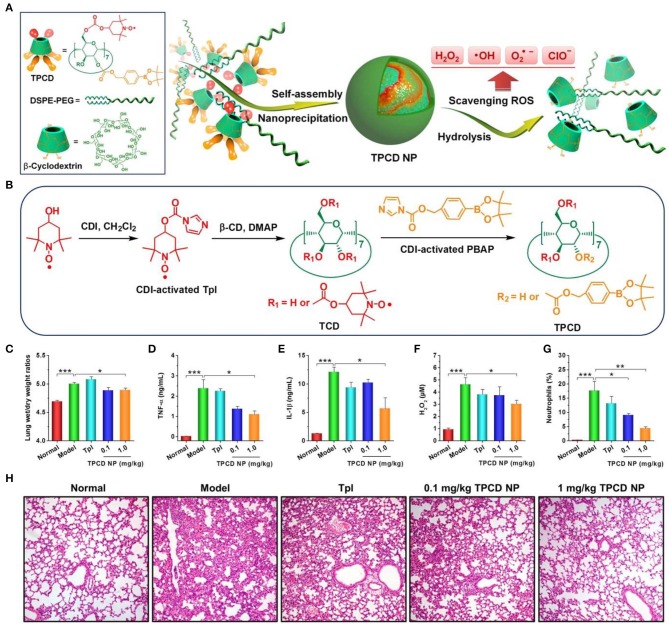
Design and preparation broad-spectrum ROS-scavenging nanoparticles for treatment of acute lung injury (ALI). **(A)** Schematic illustration of engineering of a broad-spectrum ROS-scavenging material and its nanoparticles based on functionalized β-CD. **(B)** The synthetic route of β-CD conjugated with Tempol (Tpl) and PBAP units (TPCD). CDI, 1,1-carbonyldiimidazole; DMAP, 4-dimethylaminopyridine; TCD, Tpl-conjugated β-CD; PBAP, 4-(hydroxymethyl) phenylboronic acid pinacol ester. **(C–H)** Treatment of ALI with TPCD nanoparticles (i.e., TPCD NP) in mice. **(C)** The lung wet-to-dry weight ratios after different treatments. **(D–G)** The expression levels of TNF-α **(D)**, IL-1β **(E)**, and H_2_O_2_ **(F)** in bronchoalveolar lavage fluid from mice with LPS-induced ALI and subjected to different treatments. **(G)** Quantified neutrophil counts in pulmonary tissues of ALI mice. **(H)** H&E-stained pathological sections of lung tissues. Data are mean ± standard error (*n* = 6). *P < 0.05, **P < 0.01, ***P < 0.001. Reproduced with permission from Li et al. (2018).

Nevertheless, almost all available organic nanoparticles can only eliminate limited oxygen species in ROS. To circumvent this shortcoming, our group synthesized a broad-spectrum ROS-eliminating material (i.e., TPCD), by simultaneously conjugating Tempol and phenylboronic acid pinacol ester onto β-CD (Li et al., 2018; Wang et al., 2018). TPCD can be easily produced into SOD/CAT-mimetic and size-controlled nanoparticles. The resulting TPCD nanoparticles can effectively scavenge multiple reactive species, including •${\text{O}}_{2}^{-}$, H_2_O_2_, radicals, and hypochlorite (Figures 3A,B). Compared with the corresponding nanotherapies with relative narrow-spectrum ROS-eliminating capability, TPCD nanoparticles more effectively protected macrophages from H_2_O_2_-induced apoptosis and decreased inflammatory responses *in vitro*. Consistently, TPCD nanoparticles showed superior efficacies in the animal models of acute and chronic inflammation, such as peritonitis, acute lung injury, drug-induced organ toxicity, and atherosclerosis. Importantly, preliminary *in vitro* and *in vivo* tests demonstrated the good safety profile of TPCD nanoparticles.

### Other Organic Nanoparticles

Other nanoparticles derived from small molecules such as ascorbic acid (Bhatia et al., 2019), citric acid (Wu et al., 2019), and trolox (Wattamwar et al., 2010), in combination with polymeric scaffolds, have also provided us with a powerful arsenal for antioxidant and anti-inflammatory treatment. For instance, a chemically stable antioxidant molecule, citric acid was grafted with polyvinyl alcohol (Wu et al., 2019). The obtained polymer PVA-C showed anti-apoptotic ability in stem cells, and it also upregulated the nuclear peroxisome proliferator-activated receptor γ and MnSOD by releasing citric acid.

### Drug-Loaded Nanoparticles With Antioxidant Activity

Both natural and synthetic antioxidants of small molecules have been broadly used or studied for anti-inflammatory therapies of numerous diseases associated with oxidative stress. The generally used natural antioxidants include vitamin C, vitamin E, glutathione, β-carotene, flavonoids, and curcumin, while Edaravone, lipoic acid, N-acetylcysteine, and Tempol are frequently examined synthetic compounds. The main limitations of small molecule antioxidants lie in their systemic distribution, rapid metabolism, and low retention at the diseased site, thereby resulting in low bioavailability in target tissues/cells. In this aspect, nanoparticles can protect the loaded small molecule antioxidants from hydrolysis, achieve site-specific delivery and controlled release of antioxidants at disease sites, and therefore improve their bioavailability (Table 3) (Wang et al., 2015; Kang et al., 2016; Jung et al., 2018; Larrañaga et al., 2018; Gou et al., 2019). For example, our group fabricated a SOD/CAT- mimetic nanomedicine comprising H_2_O_2_-eliminating nanoparticles derived from a PBAP-conjugated β-CD material and a free radical scavenger Tempol (Zhang Q. et al., 2016). After oral administration, this nanotherapy was able to efficiently target the inflamed colon in colitic mice, and remarkably decrease non-specific distribution, thus notably alleviating manifestations relevant to colitis, with the efficacy superior over free Tempol.

**Table 3:** Typical drug-loaded nanoparticles with antioxidant activity.

**Carrier materials**	**Loaded drugs**	**Applications**	**Evaluation models**	**Administration routes**	**References**
OxbCD	Tempol	Inflammatory bowel disease	*In vivo* in mice	Oral administration	Zhang Q. et al., 2016
Lipids	Curcumin	Sepsis	*In vitro* in RAW264.7 cells; *in vivo* in mice	Intraperitoneal injection	Wang et al., 2015
Silk fibroin	Curcumin	Inflammatory bowel disease	*In vitro* in RAW264.7 cells; *in vivo* in mice	Oral or intravenous administration	Gou et al., 2019
Vanillyl alcohol-incorporated copolyoxalate	Curcumin	Peripheral artery disease	*In vitro* in RAW264.7 cells, NIH3T3 fibroblasts, and vascular endothelial cells; *in vivo* in mice	Intramuscular injection	Jung et al., 2018
PVO	Vanillin	Ischemia/reperfusion injury	*In vitro* in RAW264.7 cells; *in vivo* in mice	Intravenous injection	Kang et al., 2016
β-Galactosidase-conjugated with anti-PECAM	CAT	Acute lung transplantation injury	*In vitro* in HUVECs; *in vivo* in rats	Intravenous injection	Kozower et al., 2003
Polyketal	SOD	Myocardial ischemia-reperfusion injury	*In vitro* in RAW264.7 cells; *in vivo* in rats	Myocardial injection	Seshadri et al., 2010
Engineered exosomes	CAT mRNA	Parkinson's Disease	*In vitro* in Neuro2A cells; *in vivo* in mice	Subcutaneous implantation with Matrigel	Kojima et al., 2018

**Figure 4:**
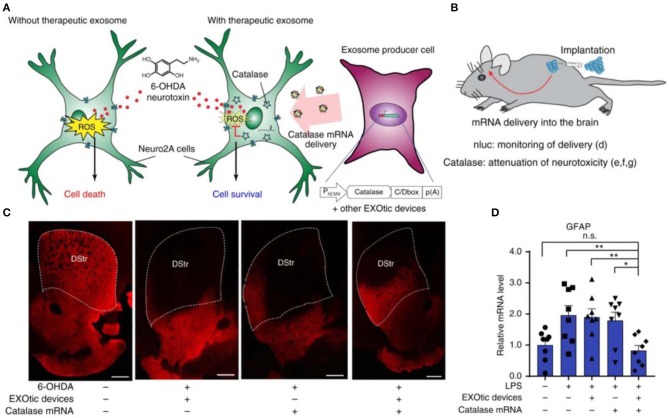
Applications of the engineered exosomes loaded with CAT mRNA in Parkinson's disease. **(A)** Schematic illustration of protection against neurotoxicity in an *in vitro* experimental model of Parkinson's disease by CAT mRNA delivery. **(B)** Engineered exosome producer cells were subcutaneously implanted with Matrigel in living mice. **(C)** Immunostaining result of tyrosine hydroxylase (TH)-positive neurons. Scale bars, 500 μm. **(D)** Attenuation of neuroinflammation caused by systemic LPS injection with catalase mRNA delivery *in vivo*. Reproduced with permission from Kojima et al. (2018). *p < 0.05, **p < 0.01; n.s., no significance.

In addition to the conventional small-molecule ROS scavengers, other biological agents, such as natural antioxidant enzymes SOD, CAT, and nucleic acids have been loaded into nanoparticles for anti-oxidative treatment (Kozower et al., 2003; Seshadri et al., 2010; Chen et al., 2013). Petro et al. constructed poly (D,L-lactide co-glycolide) (PLGA) nanoparticles separately loaded with SOD, CAT, or their combination (Petro et al., 2016). In a thromboembolic rat model, sequential administration of CAT/PLGA and SOD/PLGA nanoparticles effectively attenuated inflammatory responses, inhibited neuronal cell apoptosis, and suppressed edema formation, by protecting the blood-brain barrier (BBB) from ROS-mediated reperfusion injury. In a recent study, Kojima et al. constructed designer exosomes that can deliver CAT mRNA into target cells with high efficiency, thereby attenuating neurotoxicity and neuroinflammation in both *in vitro* and *in vivo* models of Parkinson's disease (Figure 4) (Kojima et al., 2018).

## Therapeutic Applications of Antioxidant Nanotherapies in Inflammatory Diseases

Inflammation is a protective response of the innate immune system against pathogens or irritants (Karin and Clevers, 2016). However, uncontrolled inflammation plays a fundamental role in the progress of a large number of human diseases, such as asthma, pneumonia, rheumatoid arthritis, IBD, as well as cardiovascular and neurodegenerative diseases. It has been well-documented that excessive accumulation of ROS contributes to the pathogenesis of both acute and chronic inflammation (Zhang and Kaufman, 2008). In this section, we will provide an overview of the applications of antioxidant nanomedicines in the treatment of different inflammatory diseases.

### Inflammatory Bowel Disease

Inflammatory bowel disease (IBD), including Crohn's disease and ulcerative colitis (UC), is a chronic inflammatory disorder in the gastrointestinal tract. The intestinal mucosa of patients suffering from IBD is characterized by overproduction of ROS and an imbalance of antioxidants, resulting in oxidative damage. It has been found that the mucosal ROS concentrations are 10–100 times higher in patients with IBD (Simmonds et al., 1992; Sedghi et al., 1993). Treatment with antioxidant agents can mitigate IBD in both animal models and patients (Moura et al., 2015). Unfortunately, non-specific distribution and low retention of these compounds often results in multiple side effects. So far, a number of studies have reported the construction of orally available antioxidant nanoparticles for IBD therapy with high inflamed colon retention and therapeutic effects (Vong et al., 2012; Lamprecht, 2015; Alaarg et al., 2017; Bak et al., 2018; Gou et al., 2019; Kotla et al., 2019; Li S. et al., 2019; Schilrreff et al., 2019).

**Figure 5:**
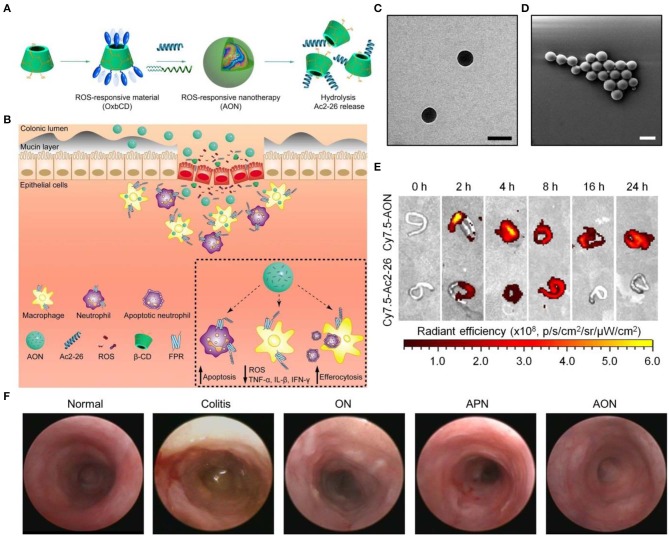
A proresolving peptide nanotherapy for site-specific treatment of inflammatory bowel disease. **(A,B)** Schematic illustration of engineering of a ROS-responsive peptide nanotherapy (AON) **(A)** and targeted treatment of colitis **(B)**. **(C,D)** TEM **(C)** and SEM **(D)** images of AON. Scale bars, 200 nm. **(E)** Selective accumulation of AON in the inflamed colons of mice with acute colitis. **(F)** Representative mini-endoscopic images of colons from colitic mice at day 7 after different treatments. ON, blank nanoparticles based on the ROS-responsive material of PBAP-conjugated β-CD (i.e., OxbCD); APN, Ac2-26-containing nanoparticles based on a non-responsive polymer PLGA. Reproduced with permission from Li C. et al. (2019).

For IBD treatment by site-specific delivery of a small-molecule antioxidant Tempol, our group fabricated a ROS-responsive nanoplatform based on a PBAP-conjugated β-CD material. The resulting SOD/CAT-mimetic nanotherapy was able to efficiently accumulate in the inflamed colon in mice and notably mitigated manifestations relevant to colitis, showing remarkable efficacies in three mouse colitis models including DSS-induced acute and chronic colitis as well as TNBS-induced colitis (Zhang Q. et al., 2016). Also, we constructed affinity nanoparticles via host-guest interactions between cyclodextrin-containing hydrophilic copolymers and Tempol. This host-guest nanotherapy exhibited desirable antioxidant and anti-inflammatory effects in macrophages and in mice with DSS-induced colitis (Xue et al., 2018). Most recently, we packaged a proresolving peptide Ac2-26 into nanoparticles derived from the aforementioned ROS-responsive material (i.e., PBAP-conjugated β-CD) to obtain a multifunctional nanotherapy, defined as AON (Figure 5) (Li C. et al., 2019). AON effectively protected Ac2-26 from degradation under gastrointestinal conditions. By oral delivery of this nanotherapy to mice with DSS-induced colitis, site-specific accumulation of AON and triggerable release of Ac2-26 in response to high levels of ROS at the inflammatory colons were successfully achieved. In mice with DSS-induced acute and chronic colitis as well as in IL-10-deficient mice that spontaneously develop colitis, oral administration of AON notably attenuated manifestations related to colitis, and significantly decreased expression of proinflammatory cytokines and oxidative stress-related molecular mediators, which was more potent than free Ac2-26 or a non-responsive control nanotherapy based on PLGA. Mechanistically, AON can effectively inhibit inflammatory responses and oxidative stress in stimulated macrophages, notably attenuate neutrophil trafficking, promote efferocytosis of apoptotic neutrophils, as well as regulate phenotypic switching of macrophages *in vitro*. *In vivo* efficacies of AON are most likely achieved by accelerating intestinal mucosal wound healing, expediting the resolution of inflammation, reshaping gut microbiota, and increasing the production of short-chain fatty acids. Moreover, oral delivery of AON showed excellent safety profile in mice.

### Cardiovascular Diseases

Cardiovascular disease is a leading cause of mortality worldwide. Increasing evidence has demonstrated that excessive ROS production is closely linked to the mitochondrial dysfunction, cell apoptosis, endoplasmic reticulum stress, and autophagy, which facilitates the occurrence and development of cardiovascular disease (Brown and Griendling, 2015). The intracellular ROS have a critical role in the pathogenesis of various cardiovascular diseases, such as atherosclerosis, myocardial hypertrophy, myocardial infarction, myocardial ischemia-reperfusion injury, and heart failure (Harrison et al., 2003). ROS in the cardiovascular system are predominantly produced by activated NADPH oxidase (NOX) which is the primary oxidase system underlying oxidative stress. NOX-derived ROS production induces activation of endothelial nitric oxide synthase (eNOS), leading to eNOS uncoupling and mitochondrial dysfunction, eventually causing sustained oxidative damage and the development of cardiovascular diseases (Zhang Y. et al., 2020).

ROS-scavenging nanotherapies have demonstrated great potential in the prevention and treatment of cardiovascular disease. They can protect cardiac progenitor cells from oxidative stress (Pagliari et al., 2012), prevent ROS-induced death of implanted mesenchymal stem cells for myocardial infarction treatment (Park et al., 2015), and eliminate excessive ROS to mitigate atherosclerosis (Chmielowski et al., 2017; Wang et al., 2018). As a typical example, our previously developed TPCD nanoparticles significantly attenuated ROS-induced inflammation and cell apoptosis in macrophages, by eliminating broad-spectrum ROS in cells (Li et al., 2018; Wang et al., 2018). In addition, TPCD nanoparticles could effectively inhibit foam cell formation in macrophages and vascular smooth muscle cells (VSMCs), by decreasing cellular internalization of oxLDL. After *i.v*. delivery, TPCD nanoparticles accumulated in atherosclerotic lesions of apolipoprotein E-deficient (ApoE^−/−^) mice, which was mainly achieved by passive targeting through the dysfunctional endothelium and translocation via inflammatory cells. In ApoE^−/−^ mice subjected to high-fat diets, treatment with TPCD nanoparticles by *i.v*. administration efficaciously inhibited the development of atherosclerosis and stabilized atherosclerotic plaques, resulting less cholesterol crystals, smaller necrotic cores, thicker fibrous caps, and lower levels of macrophages and matrix metalloproteinase-9, as compared to control drugs previously developed for antiatherosclerosis. These beneficial effects of TPCD nanoparticles were considered to be relevant to reduced systemic and local oxidative stress and inflammation. In view of the good safety profile of TPCD nanoparticles in preliminary *in vivo* tests based on different animal models (Li et al., 2018; Wang et al., 2018), this type of nanoparticles deserve further development as a potential antiatherosclerotic nanotherapy. In another study, Hao et al. developed injectable fullerenol nanoparticle-loaded alginate hydrogel, which showed excellent ROS-scavenging activity (Hao et al., 2017). This fullerenol/alginate hydrogel could serve as a cell delivery vehicle and suppress oxidative stress-mediated cell apoptosis in brown adipose-derived stem cells (BADSCs) *in vitro*, by attenuating JNK signaling pathways and activating p38 MAPK signaling pathways. Moreover, this functional hydrogel remarkably enhanced the retention and survival of implanted BADSCs in myocardial infarction zone via regulating the ROS microenvironment, thereby reinforcing therapeutic efficacy for cardiac repair.

**Figure 6:**
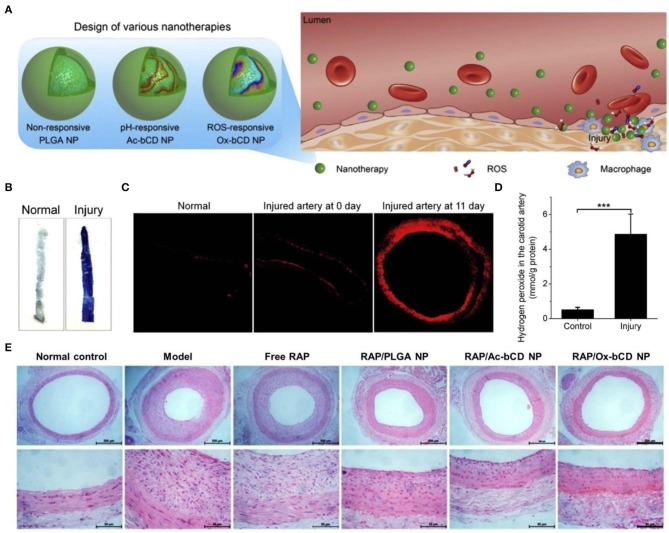
Engineering of different inflammation-responsive nanotherapies for targeted treatment of restenosis. **(A)** Design of inflammation-triggerable nanoparticles. **(B)** Evans Blue staining indicates successful establishment of injury in the carotid artery. **(C)** DHE-stained cryosections showing oxidative stress in injured carotid arteries. The image at day 0 was acquired from the sample immediately collected after injury. **(D)** The levels of hydrogen peroxide in the carotid artery with or without injury. **(E)** H&E stained histological sections of carotid arteries isolated from rats subjected to various treatments. Scale bars, 200 μm (the upper panel), 50 μm (the lower panel). PLGA NP, Ac-bCD NP, and Ox-bCD NP represents nanoparticles based on a non-responsive polymer PLGA, a pH-responsive material of acetelated β-CD, and a ROS-responsive material of PBAP-conjugated β-CD, respectively. Data are mean ± standard deviation (*n* = 3). ***P < 0.001. Reproduced with permission from Feng et al. (2016).

On the other hand, ROS-scavenging nanoparticles can serve as an effective nanoplatform for site-specific delivery of therapeutics to the sites of vascular inflammation (Seshadri et al., 2010; Wu et al., 2018; Zhang R. et al., 2020). As well-documented, substantially increased ROS levels are positively related to endothelial dysfunction and pathogenesis of restenosis after percutaneous coronary interventions (Juni et al., 2013). To develop targeting nanotherapies for restenosis, our group engineered ROS-responsive nanotherapies by loading rapamycin (RAP) into nanoparticles derived from a PBAP-conjugated β-CD material (Figure 6) (Feng et al., 2016). Thus, obtained RAP-containing nanoparticles exhibited ROS-triggerable drug release, showing significantly enhanced anti-migration and anti-proliferative effects as compared to free RAP and a non-responsive RAP nanotherapy. In addition, the ROS-responsive nanoparticles can accumulate at the injured site in the carotid artery of rats subjected to balloon angioplasty injury. In a rat model of arterial restenosis, treatment with the ROS-responsive RAP nanotherapy by *i.v*. injection more effectively attenuated neointimal hyperplasia than free RAP and a non-responsive nanotherapy. Further studies demonstrated that *in vivo* efficacy of the ROS-responsive RAP nanotherapy can be additionally improved by integrating with a pH-responsive β-CD material to afford a pH/ROS dual-responsive nanotherapy (Zhang R. et al., 2020). Of note, surface engineering of the dual-responsive nanoparticles via a peptide (KLWVLPKGGGC) targeting type IV collagen can notably increase their accumulation at injured carotid arteries, thereby potentiating *in vivo* efficacy of the dual-responsive RAP nanotherapy. Similarly, therapeutic advantages of the ROS-responsive RAP nanotherapy was demonstrated in a mouse model of atherosclerosis in ApoE^−/−^ mice (Dou et al., 2017) and an animal model of abdominal aortic aneurysm in rats (Cheng et al., 2018). In both cases, treatment with ROS-responsive nanotherapies can significantly reduce oxidative stress in diseased aortas. Consequently, ROS-responsive nanotherapies hold great potential for precision therapy of different vascular inflammatory diseases.

### Brain Diseases

Brain is an extremely metabolically active organ with low levels of antioxidant enzymes and high levels of redox-active substrates (e.g., Cu and Fe), and therefore it is especially vulnerable to oxidative stress. A growing body of evidence has substantiated that the excessive production of ROS plays a critical role in a common pathophysiology of brain diseases, such as cerebral infarction, Alzheimer's disease (AD), and Parkinson's disease (PD) (Barnham et al., 2004; Chen et al., 2011; Kim G. H. et al., 2015). Conventional antioxidant therapies cannot effectively inhibit ROS-amplified brain injury for their limited ability to cross the BBB and target the disease sites.

Antioxidant nanotherapies have been emerging as an effective strategy to overcome the BBB and to provide targeted release of different therapeutics in the impaired brain (Hu et al., 2015; Kwon et al., 2016, 2018; Liu et al., 2017). For example, Liu et al. fabricated PEG-coated melanin nanoparticles MeNPs (Liu et al., 2017). Neuroprotective and anti-inflammatory activities of MeNPs were evaluated by *in vitro* studies in Neuro 2A cells. It was found that MeNPs could decrease oxidative stress damage and inhibit CoCl_2_-induced ischemic injury, without notable effects on mitochondrial function. Further *in vivo* studies in a rat model of ischemic stroke indicated that rats pretreated with MeNPs showed less infarct area in the ischemic brain (~14%) compared with the control group (~32%). Also, MeNPs efficiently suppressed the generation of •${\text{O}}_{2}^{-}$ in brains. Consequently, MeNPs could attenuate RONS-induced inflammatory responses through suppressing the expression of typical mediators related to oxidative stress and inflammation *in vitro* and *in vivo*.

In addition, ROS-scavenging inorganic nanoparticles were used for treatment of neurodegenerative diseases. In this aspect, Kwon and coworkers constructed triphenylphosphonium (TPP)-conjugated CeO_2_ nanoparticles, for targeting the mitochondria and potential therapy of AD (Kwon et al., 2016). Such nanoparticles significantly mitigated reactive gliosis and mitochondrial morphological damage in an AD model of 5XFAD transgenic mice. In a recent study, the same group designed three different types of ceria nanoparticles, i.e., ceria, TPP-ceria, and cluster-ceria nanoparticles, to selectively scavenge intracellular, mitochondrial, and extracellular ROS, respectively (Kwon et al., 2018). Therapeutic effects of three ceria nanoparticles were compared in mice with 1-methyl-4-phenyl-1,2,3,6-tetrahydropyridine (MPTP)-induced PD. Mice treated with either ceria nanoparticles or TPP-ceria nanoparticles exhibited significantly higher levels of tyrosine hydroxylase (a hallmark of PD) and lower levels of lipid peroxidation, compared to those treated with cluster-ceria nanoparticles. This finding indicated that decreasing mitochondrial and/or intracellular oxidative stress is critical to treat PD, while elimination of extracellular ROS is not an effective strategy to prevent neurodegeneration.

### Other Inflammatory Diseases

Asthma is a chronic pulmonary disease, characterized by recurrent airflow limitation, airway remodeling, and airway hyperreactivity. Inhaling corticosteroids is the most common treatment for asthma. However, adverse effects associated with the frequent steroid administration precipitate the need for alternative therapeutics or novel delivery routes (Vij, 2011; Lim et al., 2016; Wang L. et al., 2019). Oxidative stress and inflammation play an important role in the pathogenesis of asthma. Growing evidence has indicated that nanoparticle-based anti-inflammation and antioxidant strategies are promising for the treatment of allergic airway inflammation and asthma (Fatani, 2014; Sahiner et al., 2018; Alexescu et al., 2019; Singh A. P. et al., 2019). The anti-asthmatic effects of two organic nanoparticles, i.e., BRNPs and HPOX nanoparticles with intrinsic antioxidant activity were evaluated using a murine model of asthma (Yoo et al., 2013; Kim D. E. et al., 2017). Both of them could reduce the recruitment of inflammatory cells and expression of inflammatory cytokines, thus may be used as potential nanotherapies for asthma. On the other hand, corticosteroids encapsulated in organic nanoparticles afforded more sustained therapeutic effects than free drugs. For example, a biodegradable polymer PVAX was modified to prepare dexamethasone (DEX)-loaded porous PVAX microparticles by a double emulsion method (Jeong et al., 2016). PVAX microparticles themselves remarkably reduced oxidative stress and down-regulated the expression of proinflammatory mediators such as TNF-α and iNOS. Notably, therapeutic effects of DEX-loaded porous PVAX microparticles were much better than PVAX microparticles alone, indicating a significant synergistic effect.

Acute lung injury (ALI), a heterogeneous pulmonary disease with the severe manifestation of acute respiratory distress syndrome, continues to cause high morbidity and mortality in critically ill patients (Rubenfeld et al., 2005; Sadikot et al., 2017). ALI is closely related to the systemic inflammatory response and the increased cellular ROS (Chabot et al., 1998). Cerium oxide nanoparticles were found able to reduce oxidative stress *in vitro* and *in vivo* (Arvapalli et al., 2015; Xu et al., 2016). They also exhibited protective effects against sepsis-induced ALI and radiation-induced lung injury. In mice with paraquat-induced ALI, the levels of ROS, malondialdehyde, NF-κB, phosphorylated NF-κB, TNF-α, and IL-1β were significantly reduced by porous Se@SiO_2_ nanospheres that contain Se to scavenge intracellular free radicals (Zhu et al., 2017). Also, resveratrol-loaded lipid-core nanocapsules (RSV-LNCs) were studied to ameliorate ALI via the ERK and PI3K/Akt pathways (de Oliveira et al., 2019). In addition, intrinsically bioactive nanoparticles derived from functional cyclodextrin materials TPCD and LCD that were developed by our group, were able to accumulate in the injured lungs, suppress oxidative stress, and significantly reduce the infiltration of inflammatory cells in a mouse model of ALI (Figures 3C–H) (Li et al., 2018; Guo et al., 2019). Consequently, both inorganic and organic ROS-scavenging nanoparticles can serve as a potent remedy for the treatment of ALI.

Recently, different antioxidant nanotherapies have been applied in the treatment of peritonitis, an inflammation of the peritoneum. Porfire and coauthors evaluated efficacy of three different Cu/Zn-SOD formulations in LPS-induced peritonitis (Porfire et al., 2014). PEGylated liposomes showed the most significant antioxidant and anti-inflammatory properties in this case. Ac2-26-containing nanoparticles, developed using an anti-inflammatory peptide Ac2-26 as well as diblock copolymers PLGA-PEG and collagen IV-targeting PLGA-PEG, could significantly inhibit the recruitment of polymononuclear neutrophils in a zymosan-induced peritonitis model (Kamaly et al., 2013). Our group developed H_2_O_2_-eliminating nanoparticles, based on PBAP-conjugated β-CD materials, also displayed desirable therapeutic effects in mice with peritonitis, by reducing ROS production, inhibiting neutrophil infiltration and neutrophil-induced macrophage recruitment, as well as down-regulating the expression of inflammatory cytokines and chemokines (Zhang et al., 2017).

## Concluding Remarks

Oxidative stress is at the basis of a variety of inflammatory pathologies, and therefore antioxidant nanotherapies have been extensively investigated as a new therapeutic strategy. Significant advances have been achieved in the field of antioxidant nanotherapies for different inflammatory diseases, in which ROS-scavenging inorganic nanoparticles, organic nanoparticles with intrinsic antioxidant activity, and drug-loaded nanoparticles with antioxidant activity are generally used. Indeed, extensive preclinical studies have demonstrated desirable performances of ROS-scavenging nanotherapies in different *in vitro* and *in vivo* inflammatory models. However, for the currently developed antioxidant nanotherapies, only a few of them have been intensively and systemically examined in diverse animal models. Even few translation studies have been conducted for antioxidant nanotherapies thus far.

There are still some critical challenges need to be addressed for further clinical translation of these anti-inflammatory nanotherapies. First, synthesis processes for currently developed antioxidant nanoparticles need to be optimized to produce nanotherapies with good quality control, such as highly defined structures and physicochemical characters as well as good batch-to-batch reproducibility, from the view point of preparation. The cost-efficient mass production is also a critical factor that should be carefully considered according to the benefits of different antioxidant nanoparticles and their potential applications in clinical practice. Second, both acute and long-term chronic toxicities of various antioxidant nanotherapies must be comprehensively tested, particularly for ROS-scavenging inorganic nanoparticles. The physicochemical and biological properties of nanoparticles greatly influence their interactions with the living organisms. The developed antioxidant nanotherapies or nanocarriers should be biocompatible and easy to clear by the body. Thirdly, the ROS level varies among different inflammatory disorders as well as throughout the different stages of the same inflammatory disease, which largely decide the dose and dosing frequency of antioxidant nanotherapies. The appropriate dose should control the intracellular ROS toward the beneficial therapeutic effects without causing pathological ones. These issues unfortunately remain elusive. Also, a thorough assessment of risks and benefits of antioxidative nanoparticles is a main ethical issue. The ideal antioxidative nanoparticles should maximize the well-being of patients and reduce or avoid therapy-induced side effects, thereby affording patients with the benefits outweighing the risks. With the aforementioned challenges to be resolved, we are confident that the clinical applications of antioxidant nanotherapies for the treatment of inflammatory diseases will be realize in the foreseeable future, resulting in improved safety and individualized healthcare.

## Author Contributions

C-WL and L-LL drafted the manuscript. C-WL, L-LL, and SC created the tables and figures and performed literature searches. J-XZ and W-LL revised the manuscript and edited the final draft.

### Conflict of Interest

The authors declare that the research was conducted in the absence of any commercial or financial relationships that could be construed as a potential conflict of interest.
